# Investigation of Three-Dimensional Bacterial Adhesion Manner on Model Organic Surfaces Using Quartz Crystal Microbalance with Energy Dissipation Monitoring

**DOI:** 10.1021/acsabm.2c01012

**Published:** 2023-02-20

**Authors:** Glenn Villena Latag, Taichi Nakamura, Debabrata Palai, Evan Angelo Quimada Mondarte, Tomohiro Hayashi

**Affiliations:** † Department of Materials Science and Engineering, School of Materials and Chemical Technology, Tokyo Institute of Technology, 4259 Nagatsuta-Cho, Midori-ku, Yokohama, Kanagawa 226-8502, Japan; ‡ School of Materials Science and Engineering, Nanyang Technological University, Singapore 639798, Singapore; § The Institute for Solid State Physics, The University of Tokyo, 5-1-5, Kashiwanoha, Kashiwa, Chiba 277-0882, Japan

**Keywords:** bacterial adhesion, anti-biofouling, quartz crystal microbalance, biointerface, self-assembled monolayers

## Abstract

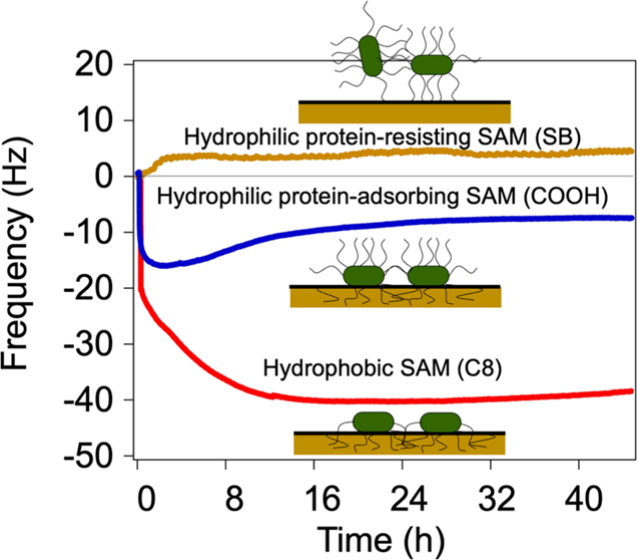

Bacterial biofilms reduce the performance and efficiency of biomedical and industrial devices. The initial step in forming bacterial biofilms is the weak and reversible attachment of the bacterial cells onto the surface. This is followed by bond maturation and secretion of polymeric substances, which initiate irreversible biofilm formation, resulting in stable biofilms. This implies that understanding the initial reversible stage of the adhesion process is crucial to prevent bacterial biofilm formation. In this study, we analyzed the adhesion processes of *E. coli* on self-assembled monolayers (SAMs) with different terminal groups using optical microscopy and quartz crystal microbalance with energy dissipation (QCM-D) monitoring. We found that a considerable number of bacterial cells adhere to hydrophobic (methyl-terminated) and hydrophilic protein-adsorbing (amine- and carboxy-terminated) SAMs forming dense bacterial adlayers while attaching weakly to hydrophilic protein-resisting SAMs [oligo(ethylene glycol) (OEG) and sulfobetaine (SB)], forming sparse but dissipative bacterial adlayers. Moreover, we observed positive shifts in the resonant frequency for the hydrophilic protein-resisting SAMs at high overtone numbers, suggesting how bacterial cells cling to the surface using their appendages as explained by the coupled-resonator model. By exploiting the differences in the acoustic wave penetration depths at each overtone, we estimated the distance of the bacterial cell body from different surfaces. The estimated distances provide a possible explanation for why bacterial cells tend to attach firmly to some surfaces and weakly to others. This result is correlated to the strength of the bacterium–substratum bonds at the interface. Elucidating how the bacterial cells adhere to different surface chemistries can be a suitable guide in identifying surfaces with a more significant probability of contamination by bacterial biofilms and designing bacteria-resistant surfaces and coatings with excellent bacterial antifouling characteristics.

## Introduction

Bacterial biofilm formation is a ubiquitous natural phenomenon.^1^ Although it is beneficial in some applications such as electricity generation^2−4^ and degradation of toxic substances in soil^5^ and water,^6^ biofilms on industrial and biomedical devices^7−9^ are detrimental to the devices’ efficiency and performance. Biofilm formation starts from the weak and reversible attachment of bacterial cells onto the surface, followed by bond maturation and secretion of polymeric substances marking the formation of stable biofilms.^10−12^ Understanding the mechanism of the early reversible stage of bacterial attachment on various surfaces is paramount in developing biofilm prevention technologies.^13−15^

The general understanding of the influence of surface chemistry on bacterial attachment is that bacterial cells adhere more to hydrophobic surfaces.^16,17^ However, some studies reported contradicting results where bacterial cells are shown to attach more on hydrophilic surfaces instead.^18,19^ Oh et al. reasoned out that the differences in the reported trends of bacterial attachment can be attributed to the ambiguity brought about by the contribution of surface topography, the variations in the introduction of bacterial cells onto the surface investigated, the removal of both adherent and nonadherent cells in the rinsing step, and the hydrodynamic nature of the bacterial suspension.^19^ In fact, observation with an optical microscope cannot solve these issues because it does not provide a detailed microscopic picture of the adhesion manner of bacteria to surfaces.

One of the devices that can address these issues is the quartz crystal microbalance with energy dissipation monitoring (QCM-D). The QCM-D is an ultrasensitive device that can monitor the adsorbed mass on a nanogram scale and the resulting adlayer viscoelasticity.^20−23^ Self-assembled monolayers (SAMs) consisting of linear-chain molecules with tunable terminal groups functionalized on atomically flat QCM sensors (surface roughness ∼1 nm) can deconvolute the influence of surface chemistry from substrate topography on bacterial attachment. Moreover, the QCM-D allows the continuous injection of the bacterial suspension to a closed module at small-volume flow rates to minimize gravitational and drying effects or to mimic *in vivo* fluid flow on biomedical devices. Moreover, the microliter precision of the fluid injection is advantageous in controlling the rinsing step, preventing the adherent cells from being dislodged from the surface.

Several QCM-D studies have shown that bacterial attachment was not governed purely by the conventional mass loading theory (inertial loading) as described by the Sauerbrey relation.^10,11,16,24−26^ Silica microparticles,^27^ diatoms,^28^ and bacterial cells have been illustrated to produce both negative and positive *f* shifts as the additional mass is being adsorbed onto the surface, which makes the mass loading challenging to interpret. Attempts to explain this complex behavior are carried out through the coupled-resonator model, which applies to micron-sized particles tethered to the surface via small connections^29^ such as bacterial cells attached to surfaces through their extracellular appendages (e.g., flagella, pili, etc.). According to this model, the QCM sensor and the adhering particle act as coupled resonators, with the latter reinforcing the oscillation of the former instead of adding an inertial resistance to slow it down.^27^ This mass loading regime is referred to as elastic loading and is governed by the stiffness of the spring-like connection between the adhering particle and the surface.^30^

Due to differences in the penetration depths (δ) of the acoustic waves simultaneously excited by the QCM, it is possible to observe both negative and positive *f* shifts in the system, with positive *f* shifts commonly occurring at high overtones (*n*) with shallower δ.^30^ Thus, the separation between *f* dominated by inertia (lower *n*) and those dominated by the link (higher *n*) can be estimated by determining the point where the *f* shifts change signs. This point is referred to as the frequency of zero-crossing or *f*_ZC_,^31^ which can give an estimation of the distances between the bacterial body and the surface through the corresponding δ at each *n*. The correlation of these estimated distances to the bacteria–substrate bond strength can provide a clearer picture of which surfaces are prone to bacterial fouling and which surfaces can potentially resist them.

In this work, we analyzed the adhesion mechanism of the flagellated *E. coli* bacteria on various SAMs using optical microscopy and QCM-D. Based on the changes in *f* and energy dissipation (*D*) of the sensor oscillation, we analyzed the mass loading and viscoelastic properties of the adhered bacterial cells with varying surface functionalities. Moreover, we elucidated the complicated stepwise adhesion process of bacterial cells to hydrophobic, hydrophilic protein-adsorbing, and hydrophilic protein-resisting SAMs, accentuating the differences brought about mainly by hydrophobic interactions, water-induced repulsion, and the presence of the interfacial water barrier. Using the QCM instrument’s ability to sense the mass loading using different overtones simultaneously acoustically, we estimated the relative distances between the bacterial cells and various surfaces, highlighting the significant role of the bacterial appendages in the adhesion process.

## Methodology

### Bacterial Cell Cultivation and Preparation

The stock solution of *E. coli* was prepared using the *E. coli* American Type Culture Collection (ATCC) 25922 strain (Difco, Detroit, MI). A certain amount of the freeze-dried bacterial cells was revived and mixed with phosphate-buffered saline (PBS) (Sigma-Aldrich) to form the *E. coli* stock solution. Using a flame-sterilized inoculating loop, the solution was streak-plated on Petri dishes containing agar media (Wako, Osaka, Japan) which were then incubated in an autoclave (Hirayama, Saitama, Japan) at 37 °C for 24 h.

The bacterial cells were harvested from the nutrient agar and then suspended in PBS. The suspension was agitated using a shaking mixer to ensure that the bacterial cells were evenly distributed throughout the adhesion buffer. Before the experiments, the turbidity of the bacterial suspension was determined using a photoabsorbance sensor (Yamato, Tokyo, Japan). With this, an absorbance of ∼0.132 a.u. corresponds to about 10^6^ bacterial cells per mL. This bacterial cell concentration was used for all microscopy and QCM-D measurements involving different monolayers.

### Fabrication of Self-Assembled Monolayers (SAMs)

AT-cut quartz crystal sensors (Biolin Scientific AB, Gothenburg, Sweden) with a fundamental resonance frequency of 5 MHz, were precleaned by subjecting them to UV-ozone for 10 min, followed by immersion in absolute ethanol (Wako, Osaka, Japan) with occasional shaking to remove the formed gold oxides.

Six types of SAMs were then fabricated through immersion of the sensors into 1 mM precursor thiol solutions in ethanol, as listed in Table 1. The immersion in absolute ethanol has to be done before the immersion in ethanolic thiol solutions to stabilize the oxides by the thiol molecules, which can potentially disturb the formation of SAMs.^32^ Finally, the SAM-modified sensors were dried with N_2_ gas shortly before the QCM-D measurements.

**Table 1:** List of SAMs Fabricated with Their Corresponding Precursor Molecules, Static Water Contact Angles, and Density Relative to Alkanethiols

notation for SAMs (based on the terminal group)	molecular formula of precursor	static water contact angle (deg)	density with respect to alkanethiols
C8^a^	HS(CH_2_)_7_CH_3_	112 (3.1)^c^	
COOH^b^	HS(CH_2_)_11_COOH	18 (4.4)^c^	95 (2.0)^c^
NH_2_^b^	HS(CH_2_)_11_NH_2_	35 (5.4)^c^	97 (2.2)^c^
OH^a^	HS(CH_2_)_11_OH	17 (2.6)^c^	98 (1.4)^d^
OEG^c^	HS(CH_2_)_11_(OCH_2_CH_2_)_3_OH	32 (4.3)^c^	86 (2.9)^c^
SB^a^	HS(CH_2_)_11_N^+^(CH_3_)_2_CH_2_SO_3_^–^	39.6 (3.9)^c^	59.5 (0.72)^d^

a Purchased from Sigma-Aldrich.

b Purchased from ProChimia Surfaces.

c Numbers in parentheses are standard deviations; *N* = 5.

d *N* = 12; Data reproduced from Hayashi et al.^33^

### Optical Microscopy

The gold-coated quartz sensors functionalized with different SAMs were immersed in the prepared bacterial suspension for the same duration as the QCM-D experiment described in the next section. Before taking the microscopy images, the sensors were rinsed to remove loosely bound bacterial cells by slowly flooding the dish with PBS solution. Then, the bright-field microscope images of the bacterial cells adhering to the substrates were taken using an inverted optical microscope (Nikon Eclipse TE2000-U, Tokyo, Japan) in transmitting configuration. At least three micrographs were taken for each sample, and the measurements were repeated on at least two occasions. The number of bacterial cells per unit area was counted using the ImageJ software.^34^

### QCM-D Measurements

**Figure 1:**
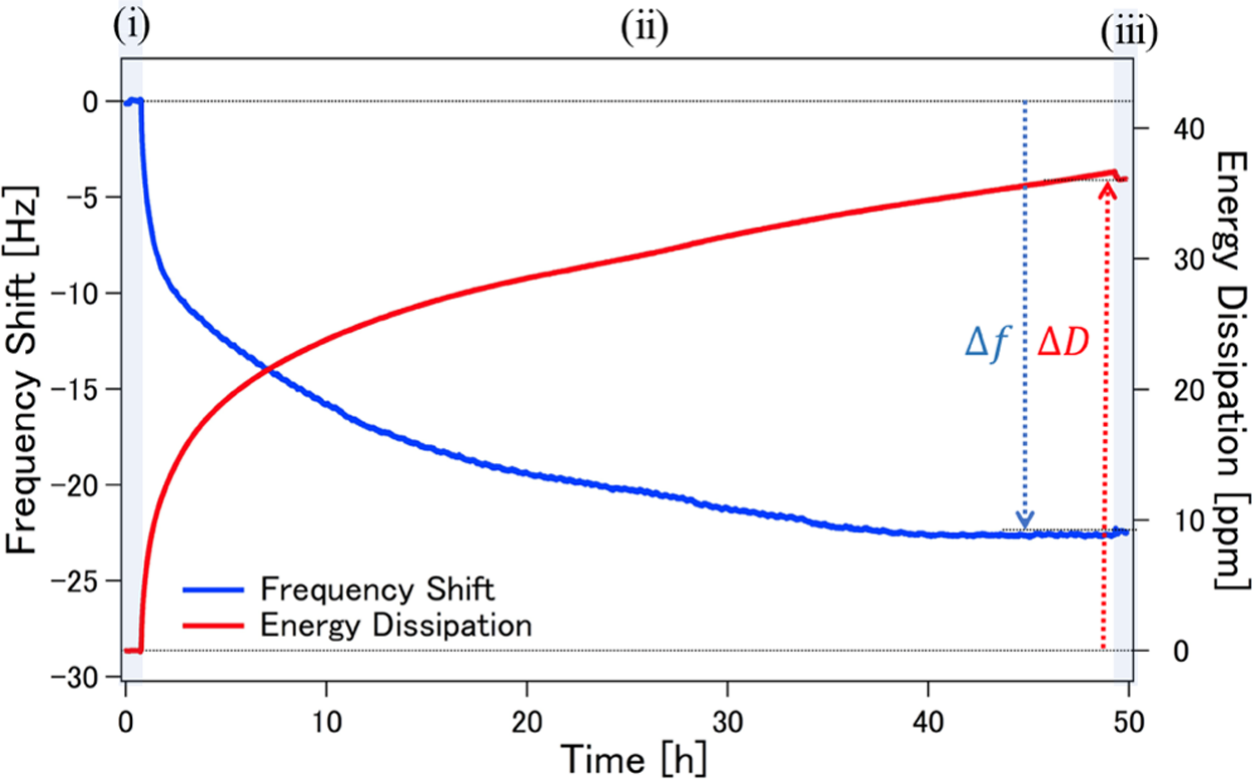
Typical evolution of frequency and energy dissipation shifts in bacterial adhesion. Regions (i), (ii), and (iii) represent baseline establishment in PBS, contact with bacterial solution, and rinse with PBS, respectively. Changes in frequency (Δ*f*) and dissipation (Δ*D*) were calculated as the difference between (i) and (iii), as shown by arrows.

The functionalized gold-coated quartz sensors were placed inside the flow modules of the commercial QCM-D setup (Biolin Scientific AB, Gothenburg, Sweden). The solutions were injected into the flow modules using a peristaltic pump (Minato, Tokyo, Japan) and the flow rate was kept constant at 50 μL/s throughout the experiment. The frequency (*f*) and dissipation (*D*) shifts were recorded through the time course of (i) establishing baseline in the PBS environment, (ii) introduction of the *E. coli* solution up to stabilization of signals, and finally, (iii) rinsing with PBS until signals had stabilized again, as shown in Figure 1.

The changes in *f* (Hz) and *D* (ppm) were defined as the differences in their respective values between time courses (i) and (iii) and were monitored for six different overtones, *n* = 3, 5, 7, 9, 11, and 13. For the real-time monitoring of *f* shifts and the description of the changes in the viscoelasticity of the layers, we chose *n* = 3 and 13 to observe the adhesion manner farthest and nearest to the sensor surface, respectively. After every measurement, the solution flow path was cleaned with 2% sodium dodecyl sulfate, SDS (Wako, Osaka, Japan) followed by pure water.

## Results and Discussion

### Adhesion Observed by Optical Microscopy

**Figure 2:**
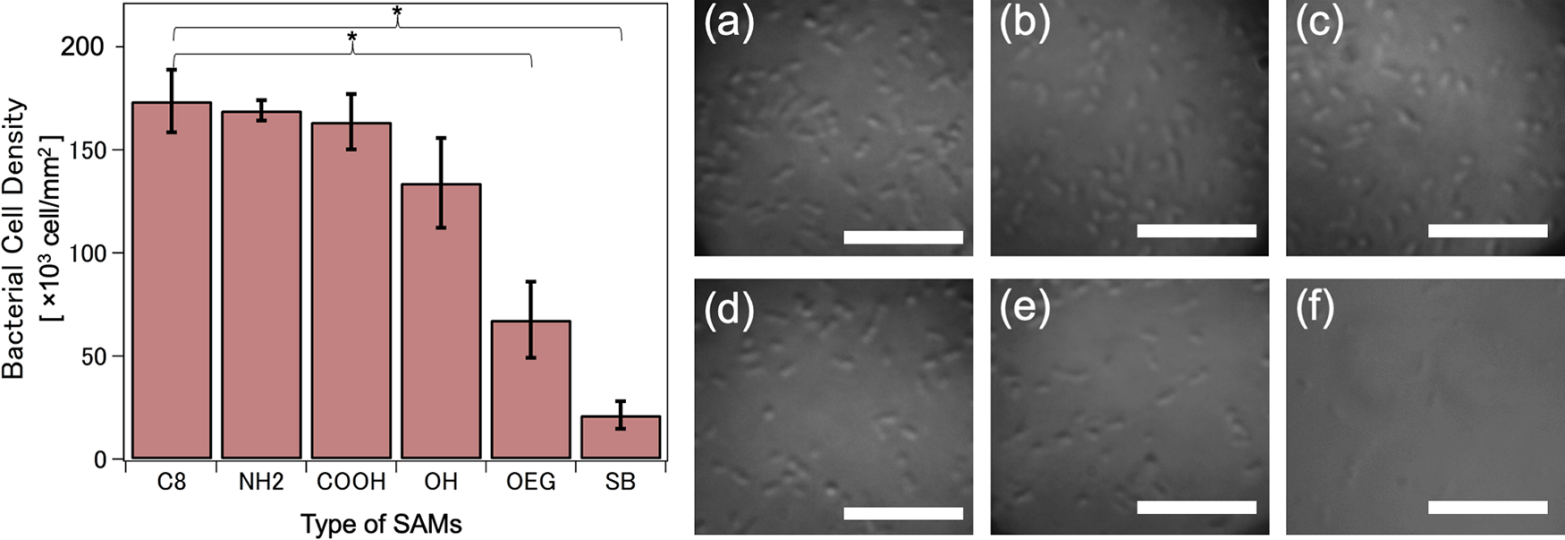
(Left) Bar graph showing bacterial cell density (cells/mm^2^) on different SAMs. Error bars denote standard deviation (*N* = 6), and asterisks (*) denote statistical significance (*p* < 0.001). (Right) Representative micrographs of bacterial attachment for (a) hydrophobic C8 SAMs; hydrophilic protein-adsorbing (b) NH_2_, (c) COOH, and (d) OH SAMs; and hydrophilic protein-resisting (e) OEG and (f) SB SAMs. Scale bars denote 10 μm.

Figure 2 shows the actual surface density of bacterial cells adhering to the different SAMs. The hydrophobic protein-adsorbing methyl-terminated (C8) SAMs, and hydrophilic protein-adsorbing amine (NH_2_)-, carboxy (COOH)-, and hydroxy (OH)-terminated SAMs exhibited significant bacterial adhesion per unit area. On the other hand, the bacterial cell density decreased significantly (*p* < 0.001), for the hydrophilic protein-resistant oligo-ethylene glycol (OEG)- and sulfobetaine (SB)-terminated SAMs relative to the hydrophobic SAMs.

The hydrophilic protein-resistant OEG and SB SAMs exhibited bacterial adhesion at low densities, and thus can potentially inhibit bacterial adhesion. These SAMs are known to have a structured water layer at the interface, which plays a significant role in protein-fouling resistance.^33,35−37^ Detailed analyses about the progression of bacterium–substratum interactions, bond maturation, and bacterial cell adlayer viscoelasticity in relation to changing the surface chemistry were carried out using QCM-D as described in the succeeding sections.

### Adhesion Kinetics of Bacterial Cells on SAMs

**Figure 3:**
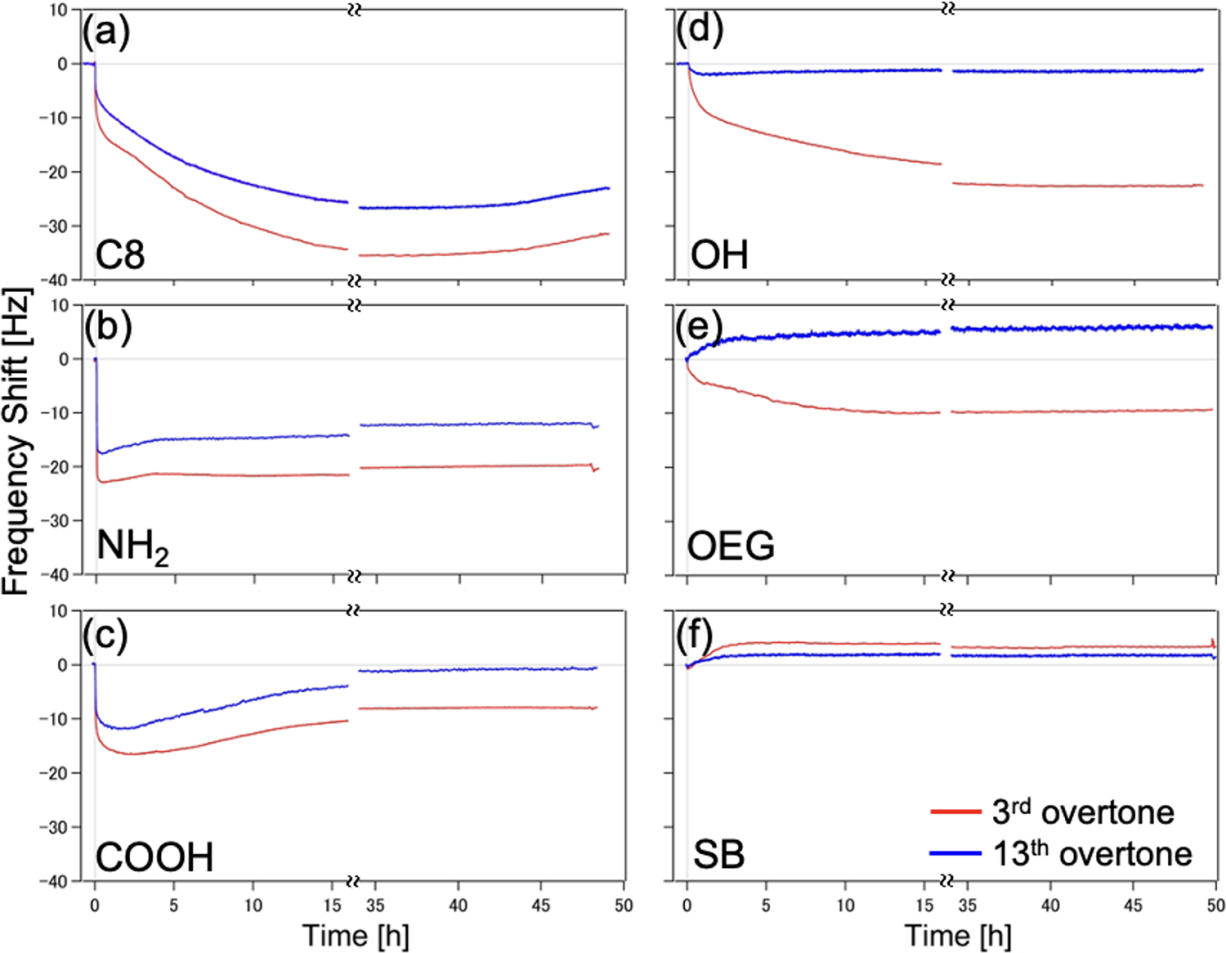
Time progression of the frequency shifts at *n* = 3 (red) and 13 (blue) on (a) hydrophobic C8 SAMs; hydrophilic protein-adsorbing (b) NH_2_ SAMs, (c) COOH SAMs, and (d) OH SAMs; and hydrophilic protein-resisting (e) OEG SAMs and (f) SB SAMs.

Figure 3 shows the time profiles of the frequency (*f*) shifts of the QCM sensors (*n* = 3 and *n* = 13) functionalized with different SAMs during the 48–50 h experimental runs. The variation in the *f* shifts at different *n* is due to the differences in the penetration depths (δ) of the acoustic shear waves. Since the δ of *n* = 3 is deeper than that of *n* = 13, the sensor can detect more mass, and thus, larger *f* shifts are expected.

The hydrophobic C8 SAMs (Figure 3a) showed a sudden drop in *f* shift (−12 Hz for *n* = 3 and −6 Hz for *n* = 13) in the first few minutes after the injection of the bacterial suspension, followed by a gradual and continuous decline after a few hours. This implies a rapid initial attachment followed by continuous accumulation of bacterial cells onto the hydrophobic surface. Then, constant negative *f* shifts (−35 Hz for *n* = 3 and −25 Hz for *n* = 13) were observed between 15 and 45 h, which can be attributed to the saturation of the surface with bacterial cells. The last five hours exhibited a gradual decrease in the magnitude of the *f* shift, which signifies the removal of portions of the bacterial biofilm from the surface.

The hydrophilic protein-adsorbing NH_2_ (Figure 3b) and COOH (Figure 3c) SAMs also exhibited a rapid decrease in *f* shift a few minutes after the injection of the bacterial suspension, with the NH_2_ SAMs exhibiting the largest initial drop in *f* (−22 Hz for *n* = 3 and −17 Hz for *n* = 13). Oh et al. reasoned out that the electrostatic interaction is evident between the positively charged amine groups and the predominantly negatively charged bacterial membrane resulting in substantial bacterial adhesion.^19^ However, the magnitude of the initial *f* shifts for both NH_2_ and COOH SAMs declined after reaching a maximum value. This decrease signifies a loss in the adhered bacterial mass at the initial stages of bacterial attachment and gives deeper insights into the stability of the biofilm buildup on charged hydrophilic surfaces.

Interestingly, the hydrophilic protein-resisting OEG SAMs (Figure 3e) displayed negative *f* shifts at *n* = 3 and positive *f* shifts at *n* = 13, while the hydrophilic protein-resisting SB SAMs (Figure 3f) showed positive *f* shifts regardless of *n*. Interpretation of these positive *f* shifts was made using the coupled-resonator model.^25,30^ This model suggests that the micron-sized bacterial cells attach to the sensor using their appendages. The clinging configuration reinforces the oscillation of the sensor instead of slowing it down, thereby resulting in positive *f* shifts. This will be further discussed in detail in the succeeding sections.

**Figure 4:**
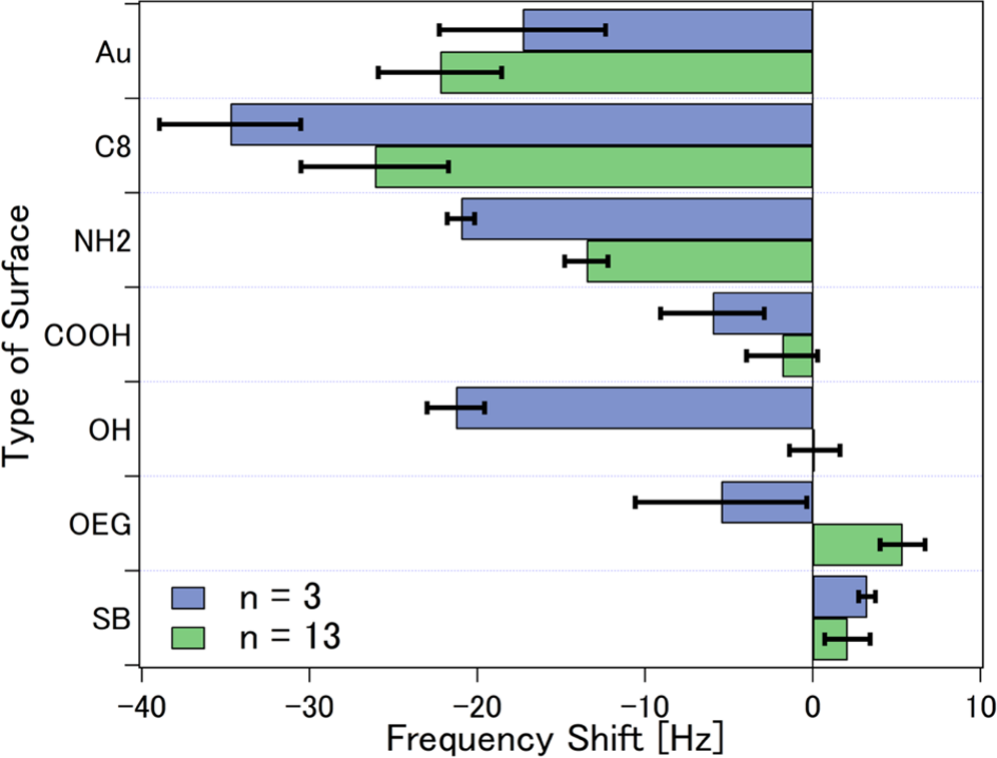
Equilibrium f shifts (*n* = 3 and 13) on bare gold surface, hydrophobic C8 SAMs; hydrophilic protein-adsorbing NH_2_ SAMs, COOH SAMs, and OH SAMs; and hydrophilic protein-resisting OEG SAMs, and SB SAMs. Error bars denote standard deviation.

The equilibrium *f* shifts after rinsing the sensors with the buffer solution for *n* = 3 and 13 were then calculated as shown in Figure 4. The largest equilibrium *f* shifts occurred on the hydrophobic C8 SAMs, which is consistent with the results of the microscopy images. Even though the protein-adsorbing NH_2_ and COOH SAMs exhibited the largest *f* shifts at the onset of bacterial injection, their *f* shifts declined continuously over time resulting in smaller equilibrium shifts compared to those found in the hydrophobic SAMs. This means that in the long run, the charged hydrophilic NH_2_ and COOH SAMs can potentially show better low-fouling performance against bacterial biofilm formation compared to their hydrophobic SAM counterpart despite experiencing more significant bacterial adhesion at the first few minutes of bacterial injection.

Friedlander et al. also reported that the equilibrium adhesion of flagellated *E. coli* on hydrophobic surfaces is greater than that on hydrophilic surfaces.^17^ They reasoned that strong hydrophobic interactions are evident between the hydrophobic flagellar filaments and the hydrophobic surfaces which favors continuous bacterial attachment over time. On the other hand, the bacterial appendages only bind loosely to the hydrophilic surfaces and result in a more reversible attachment instead.

The hydrophilic protein-resisting OEG SAM exhibited negative *f* shift at *n* = 3 (−5.5 ± 5.1 Hz) and positive *f* shifts at *n* = 13 (5.4 ± 1.3 Hz) while the SB SAM registered positive *f* shifts at both overtones (3.2 ± 0.5 Hz for *n* = 3 and 2.7 ± 1.4 Hz for *n* = 13). Using a coupled-resonator model, Olsson et al. explained that positive *f* shifts occur when the adhering micron-sized mass is attached to the surface through a small connection,^30^ e.g., bacterial cells attached to the surface through extracellular appendages. They explained that this connection acts as an elastic spring and instead of slowing down the sensor oscillation, it exerts a restoring force onto the vibrating QCM crystal. This restoring force increases the sensor’s overall oscillation frequency; hence, positive *f* shifts were observed.

In this study, it is proposed that the bacterial attachment to the hydrophilic protein-resistant SAMs (OEG and SB) follows the coupled-resonator model. Even though there exists a layer of structured interfacial water surrounding the protein-resisting SAMs,^33,35^ the bacterial appendages possess sufficient vibrational energy to break through this barrier and ultimately attach to the sensor surface.^17^ The bacterial body, however, could not penetrate through the water barrier and thus, it remains afloat in the bulk liquid. We speculate that this clinging configuration is the reason behind the observed positive *f* shifts.

Marcus et al. also reported positive *f* shifts in their study of the adhesion of *P. aeruginosa* onto silica and poly(vinylidene fluoride) surfaces.^16^ They also attributed the positive *f* shifts to the elastic spring-like connection (appendages) between the bacteria and the sensor surface, reasoning that this connection counterbalances the added inertia of the adhering mass and thus results in positive *f* shifts. Similarly, van der Westen et al. reported positive *f* shifts in fibrillated streptococcal *S. salivarius* strain, while purely inertial (negative) *f* shifts were recorded for the bald streptococcal counterpart.^25^ This study strengthened the claim that bacterial appendages are playing a key role in bacterial attachment, especially to protein-resistant surfaces.

**Figure 5:**
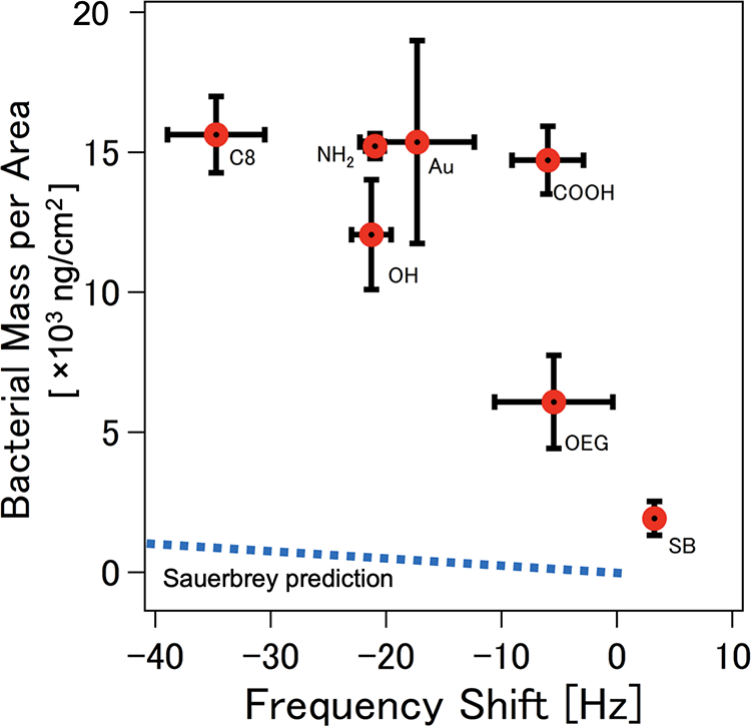
Plot of bacterial cell mass derived from the experimentally observed cell density from optical microscopy data against the QCM’s equilibrium frequency shift (*n* = 3) for the hydrophobic SAM, hydrophilic protein-adsorbing SAMs, hydrophilic protein-resisting SAMs, and bare Au surface. Vertical and horizontal error bars denote standard deviation for the derived areal bacterial mass and the frequency shifts, respectively. The blue dotted line represents the prediction of bacterial cell mass from the f shifts using the Sauerbrey relation (*n* = 3).

We then clarified if the conventional mass loading theory (Sauerbrey relation) can be used to predict the actual bacterial cell attachment in this study. To show this, we plotted the adhered bacterial mass, calculated from the observed bacterial density, against the QCM’s equilibrium *f* shift for all SAMs as shown in Figure 5. We included the function of bacterial mass predicted by the Sauerbrey relation for comparison. The derivation of this function is summarized in the Supporting Information (Section S1).

It is evident that the bacterial attachment is largely underestimated by the relation, which is probably due to the thickness and soft nature of the adhered bacterial cell layers, extracellular polymeric substances (EPS), and the volume of water trapped in the bacteria–substrate and bacteria–bacteria interfaces. The relation is only applicable to rigid adlayers characterized by nonspreading frequency profiles which were not observed in our measurements. Furthermore, the relation failed to explain the occurrence of the positive *f* shifts found on the protein-resisting SAMs, especially at higher overtone numbers.

### Viscoelasticity of Layers Analyzed via Energy Dissipation

The ratios between the energy dissipation and frequency shifts (Δ*D*/Δ*f*) values remove the dependence of energy dissipation on the actual mass of adhered material and thus will give a more accurate interpretation of bacterial layer rigidity. The hydrophobic C8 SAMs exhibited the least magnitude of Δ*D*/Δ*f* (0.34 ± 0.12 ppm/Hz for *n* = 3) implying that bacterial cells create rigid layers and stronger bonds with the hydrophobic surface compared to the other SAMs. Berne et al. explained that upon extended surface contact, the surface structures of the *E. coli* bacteria undergo rearrangement wherein hydrophobic residues from their fibrillar adhesins promote hydrophobic interactions,^38^ resulting in tighter surface interactions and thus rigid bacterial layers. The small Δ*D*/Δ*f* also agrees with the study of Olsson et al. wherein they explained that a decrease in energy dissipation is an indicator of the removal of water from the bacterium–substratum interface as the bacterial cells get closer to the sensor surface to form a rigid adlayer.^11^

On the other hand, relatively larger Δ*D/*Δ*f* values were observed for the hydrophilic NH_2_ SAM (0.51 ± 0.05 ppm/Hz for *n* = 3) and COOH SAM (1.01 ± 0.55 ppm/Hz for *n* = 3), which implies that the bacterial cell layers formed are less rigid compared to those in the hydrophobic SAMs. The water trapped at the bacteria–substrate and bacteria–bacteria interfaces were presumed to be the reason for the more significant Δ*D*/Δ*f*. Marcus et al. explained that the energy dissipation will be larger if water is trapped in the interface since the viscous shear will increase due to viscous friction of the liquid gap between the surface and the attached body.^16^

### Bacterial Adhesion Analyzed Based on the Physicochemical Property of Surfaces

**Figure 6:**
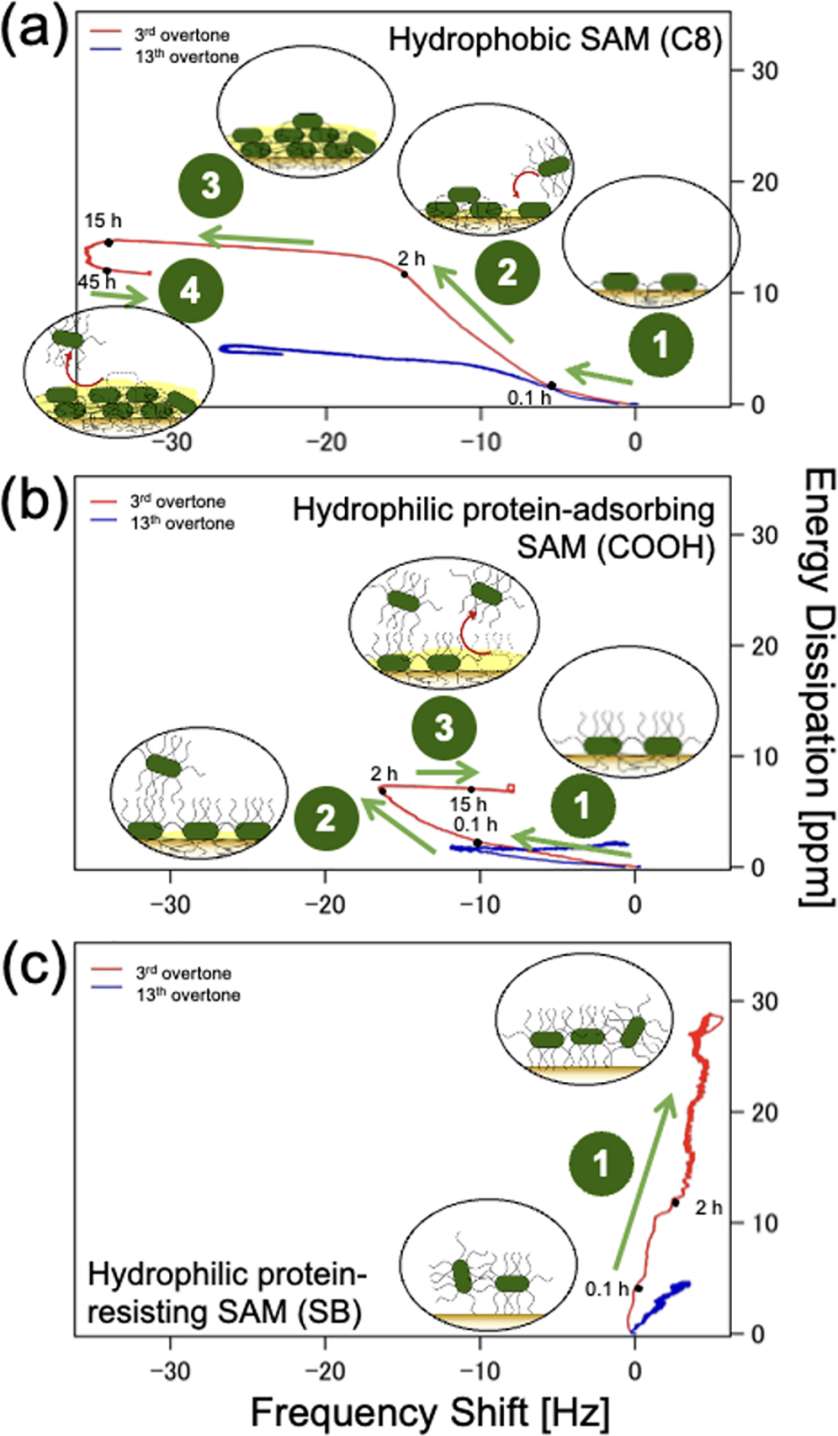
*D* versus *f* shift plot at *n* = 3 (red) and *n* = 13 (blue) showing the time progression of bacterial adhesion for (a) hydrophobic (C8) SAMs; (b) representative hydrophilic protein-adsorbing (COOH) SAMs; and (c) representative hydrophilic protein-resisting SB SAMs. Time stamps were shown for the *D* versus *f* shift plot at *n* = 3 to aid time visualization.

The nonuniform distribution of cells on the sensor surface and the uneven contribution of each cell to the *f* and *D* shifts can be minimized by presenting a plot of *D* against the *f* shifts.^16^Figure 6 gives the progression of the changes in the viscoelastic property of the adhered bacterial cell layers for representative SAMs. The *D* versus *f* plots for all SAMs are shown in Figure S2. Identical shapes of the curves at the different overtones were observed, with the adhesion kinetics at the lower overtone being more pronounced since the acoustic wave reaches larger penetration depths.

For the hydrophobic C8 SAMs (Figure 6a), the bacterial adhesion process is initially characterized by a linear plot of negative *f* shift and positive *D* shift. This contact initiation step (a1) happened in a short interval of time (∼0.1 h) after the injection of the bacterial suspension into the flow module. For the hydrophobic surface, the formation of the bacterial biofilm was initialized by the hydrophobic bacterial appendages, which readily adhere to the hydrophobic surface via strong hydrophobic interactions.^39^

The succeeding attachment up to the second hour (a2) features a steeper slope, which entails that the energy dissipation increase is more pronounced than the adhesion of additional bacterial mass. This steeper slope signifies the formation of a softer and weakly bound layer of bacterial cells clustered on top of one another. Then, a near-constant dissipation shift was observed until the 15th hour (a3), signifying that the change in energy dissipation is becoming negligible even though more bacterial mass is being deposited. This indicates the irreversible biofilm formation wherein the extended surface contact between the hydrophobic appendages and the hydrophobic surface resulted in tighter surface interactions.^38^ From 15 to 45 h, minimal changes were seen in the *f* and *D* shifts which evidence the formation of a stable EPS-protected biofilm. Around the 45th hour, a “hook” feature characterized by a slight decrease in *D* shift and a noticeable shift in *f* toward the positive direction (a4) was observed. This means that some cells detached from the layer farthest from the sensor surface as they probably returned to their planktonic (free-swimming) state.

For the hydrophilic protein-adsorbing COOH (Figure 6b) SAM, the first two steps of contact initiation (b1) and bacterial cell layer buildup (b2) observed in hydrophobic SAMs were also present. After two hours, a plateauing of the energy dissipation in the direction of a positive *f* shift (b3) occurred. This means that some of the rigid bacterial layers were removed from the surface, thus producing *f* shifts in the positive direction.

Friedlander et al. explained that the bacterial appendages were not “zipped” onto the hydrophilic surfaces, and most of the filaments remain unbound.^17^ This means that even though the initial bacterial attachment to hydrophilic SAMs is greater than that of the hydrophobic SAMs (up to the first two hours), bacterial cluster formation on hydrophilic SAMs would be less probable due to the steric hindrance imposed by the free bacterial appendages. More importantly, the *f* shift toward the positive direction signifies the weaker bonding between the bacterial cells and the surface and implies a more reversible bacterial attachment even at the latter stages of the bacterial adhesion process.

For the hydrophilic protein-adsorbing SB SAMs (Figure 6c), a trend characterized by positive *f* shift and large *D* shift was observed. As mentioned, positive *f* shifts occur when the bacterial cell attaches to the sensor only through their appendages.^17^ The interfacial water layer between the bacterial cells and the SAMs, as well as how the bacterial cells cling to the surface, are the probable reasons for the large *D* shift.

### Cross-Sectional View of Bacteria–Substrate Interface Estimated from the Overtone Dependence of *f* Shifts

**Figure 7:**
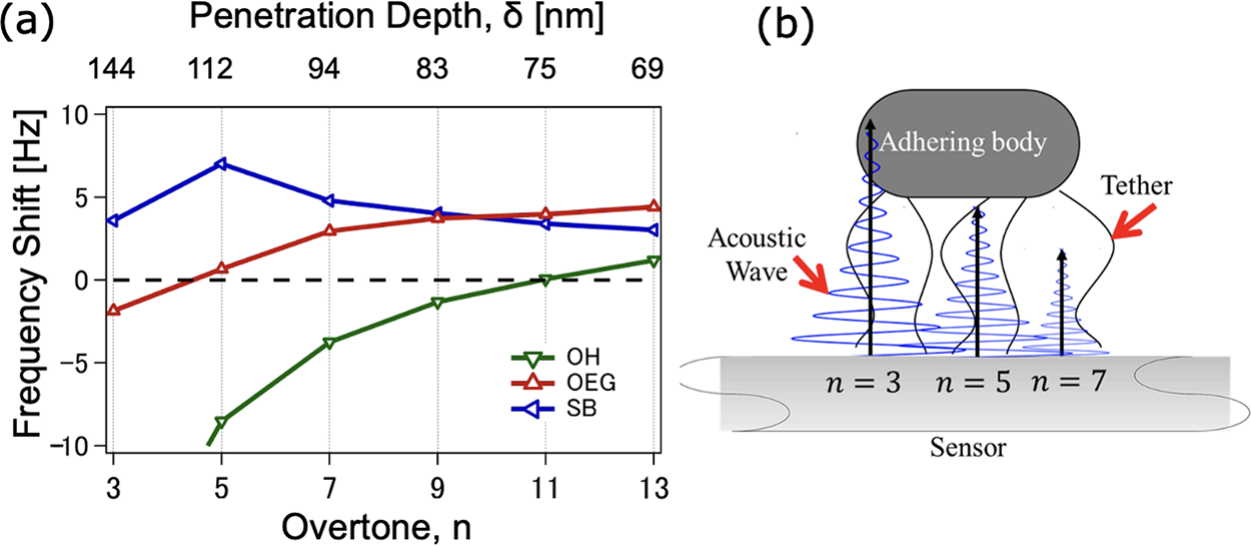
(a) Overtone dependence of the equilibrium *f* shifts for the SAMs that showed positive *f* shifts. (b) Visualization of the δ of the evanescent shear wave for the 3rd, 5th, and 7th overtones superimposed on the adhering body (bacterial cell) tethered to the sensor via small links (bacterial appendages).

The hydrophobic C8 SAMs and the hydrophilic NH_2_ and COOH SAMs have shown negative *f* shifts at all *n*, which means that the mass loading for these surfaces is governed purely by the inertial regime. Both OH and OEG SAMs manifested positive *f* shifts at higher *n*, while the SB SAMs showed positive *f* shifts at all *n* (Figure 7a). The overtone dependence of the *f* shifts for all SAMs is summarized in Figure S3.

The resonance frequency corresponding to an *n* is inversely related to the acoustic wave’s δ, which means that large *n* corresponds to shallow acoustic sensing, i.e., areas very close to the sensor surface. We exploited this information to estimate at which resonance frequency, called the frequency of zero-crossing (*f*_ZC_), the *f* shifts change the sign from negative to positive. For example, in the case of OEG SAMs, the *f*_ZC_ is located between *n* = 3 and 5 with corresponding δ of 144 and 112 nm, respectively.

The *f*_ZC_ can be interpreted as the boundary between the regimes governed by inertia (direct contact of bacterial mass to sensor surface) and that governed by the elastic spring-like connection attributed to the bacterial appendages. It can also be the basis of the critical δ value marking the distance from the sensor where the acoustic wave can still reach the bacterial body (Figure 7b). To show this, we determined the *f*_ZC_ for those SAMs that exhibited both positive and negative *f* shifts (i.e., OH and OEG). The *f*_ZC_ for the SB SAMs is out of scope in the current experimental setup.

**Figure 8:**
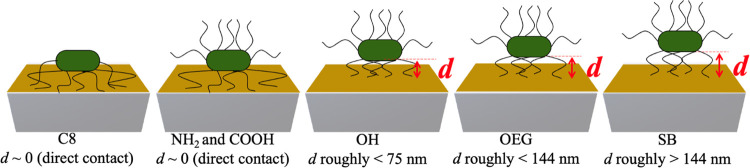
Illustration of SAM–bacteria interface showing the estimated distances d of the bacterial cell body from the different SAM surfaces.

Figure 8 shows the summary of the estimated distances (*d*) of the bacterial body from the functionalized sensor surface as deducted from the *f* shifts and the *f*_ZC_ values shown in Figure 7a. For the SAMs that showed negative *f* shifts at all overtones (C8, COOH, and NH_2_), the inertial mass loading regime dominates, and thus, direct contact between the bacterial cell and the surface is proposed. The significant difference between the mode of attachment of the bacterial cells toward the hydrophobic and hydrophilic SAMs may be described from the configuration of the bacterial appendages. For the hydrophobic SAMs, most of the flagellar filaments are spread like a carpet and zipped onto the surface, while for the hydrophilic SAMs, most of the appendages are free-moving in the bulk liquid.^17^

Next, we observed that the *f*_ZC_ for the OH SAMs lies around *n* = 11, which corresponds to δ equal to 75 nm. This means that the acoustic wave’s δ of *n* < 11 (δ > 75 nm) can still reach the bacterial body and, thus, register a negative *f* shift (inertial loading). The opposite is true for *n* > 11 (δ < 75 nm), which can only penetrate through the bacterial appendages and thus register a positive *f* shift (elastic loading). Therefore, we report the estimated *d* of the bacterial body from the surface to be less than 75 nm. In a similar analogy, the *f*_ZC_ of the OEG SAMs lies between *n* = 3 (δ = 144 nm) and *n* = 5 (δ = 112 nm); thus, we report the estimated *d* to be less than 144 nm. Finally, since the SB SAMs showed positive *f* shifts at all *n*, we note that the *d* of the bacterial body from the surface is more than the δ of the lowest *n* from our data set, i.e., greater than 144 nm.

Bacteria–surface distances have been evaluated by total internal reflection microscopy (TIRM).^40,41^ Among them, Vigeant et al. monitored the distance of individual bacteria on various surfaces. In the case of irreversible adhesion, the distance was several nm. On the other hand, in the case of reversible adhesion, the distance was 30–40 nm. These findings are quantitatively in agreement with our results. It should be noted that the distance evaluated by TIRM is smaller than that by QCM-D. The main reason could be the difference in their sampling depths. The sampling depth of TIRM is about 100 nm. Therefore, TIRM selectively measures detectable individual bacteria cells that stay in the vicinity of the surface. In the case of QCM-D, the sampling depth (decay length of the acoustic wave) is 144 nm, and in addition, QCM-D measures the ensemble of bacteria. Considering the length of the appendages (tens of nm to several microns)^38^ used by the bacterial cell to anchor to the surface, it is reasonable to observe larger distances in the case of QCM-D.

These estimated distances can provide a quantitative picture of the attachment manner of bacterial cells assisted by their appendages. This estimation applies especially to hydrophilic protein-resistant surface chemistries surrounded by a layer of structured interfacial water responsible for the SAMs’ protein antifouling property.^33,35^ Furthermore, these distances may be correlated to the strength of the bacterium–substratum bond, providing insights into which surfaces are vulnerable to bacterial fouling and which surfaces can potentially resist them.

## Summary and Conclusions

We have performed optical microscopy and QCM-D experiments to investigate the adhesion of *E. coli* on hydrophobic (C8), hydrophilic protein-adsorbing (NH_2_, COOH, and OH), and hydrophilic protein-resisting (OEG and SB) SAMs. From the microscopy images, it was found that the bacterial cells adhere to the hydrophobic and hydrophilic protein-adsorbing SAM. In contrast, a less significant bacterial attachment was observed for the hydrophilic protein-resisting SAMs. The QCM-D data also revealed that bacterial cells adhere strongly to hydrophobic SAMs and weakly to hydrophilic SAMs.

We also investigated the relationship between the *D* and *f* shifts for all SAMs and qualitatively described the progression of bacterial attachment and the resulting layer viscoelasticity. Through this, we showed the variation in the behavior of the *E. coli* bacteria in the context of particle adhesion stressing that the bacterial appendages play a crucial role in initiating contact with the surface, building bonds, and creating elastic spring-like connections to the surface, which caused positive *f* shifts. Finally, we investigated the overtone dependence of the *f* shifts, and through the *f*_ZC_, we estimated the distances of the bacterial cell bodies from the surfaces of different SAMs. The estimated distances can be correlated to the strength of the bacterium–substratum bonds brought about by the influence of the functionality of the surface. The tendency of bacterial cells to adhere to different surface chemistries is valuable in understanding the onset of bacterial biofilm formation. This study provides a practical guide in determining which surfaces are prone to bacterial fouling and gives insights into designing bacterial biofilm prevention strategies for numerous biomedical and industrial devices.

## Supporting Information Available

Derivation of theoretical bacterial adhesion from the Sauerbrey equation; time progression of bacterial layer viscoelasticity from *D* vs *f* plots; and overtone dependence of the equilibrium *f* shifts (PDF)
