# Supplementary material for: Investigation of Three-Dimensional Bacterial Adhesion Manner on Model Organic Surfaces Using Quartz Crystal Microbalance with Energy Dissipation Monitoring
Source: ACS Appl Bio Mater. 2023 Feb 20;6(3):1185–94. doi: 10.1021/acsabm.2c01012 (PMC10031553; doi:10.1021/acsabm.2c01012)
Supplement: Supplementary file 1 — mt2c01012_si_001.pdf [file mt2c01012_si_001.pdf]

Supporting Information

Investigation of three-dimensional bacterial adhesion manner on model organic surfaces using quartz crystal microbalance with energy dissipation monitoring

Glenn Villena Latag[1], Taichi Nakamura[1], Debabrata Palai[1], Evan Angelo Quimada Mondarte[1,2,], and Tomohiro Hayashi[1,3,*]*

[1] Department of Materials Science and Engineering, School of Materials and Chemical Technology, Tokyo Institute of Technology, 4259 Agastache, Midori-ku, Yokohama, Kanagawa 226-8502, Japan

[2] School of Materials Science and Engineering, Nanyang Technological University, Singapore 639798

[3] The Institute for Solid State Physics, The University of Tokyo, 5-1-5, Kashiwanoha, Kashiwa, Chiba 277-0882, Japan

*E-mail: tomo@mac.titech.ac.jp and evanangelo.mondarte@ntu.edu.sg

S1 Derivation of theoretical bacterial adhesion from the Sauerbrey equation

In the conventional mass loading theory described by Sauerbrey,[S1] a negative frequency shift from the QCM-D data is linearly proportional to the mass of the particles adhering to the surface (inertial loading). In equation form, it is represented as:

$$\Delta m = -C\frac{\Delta f}{n}$$

where Δm is the mass of the particles adhering to the sensor surface, C is the mass sensitivity constant, which is an intrinsic property of the quartz crystal, Δf is the change in the resonant frequency, and n is the harmonic number. For a 5 MHz AT-cut quartz crystal, C is equal to 17.7 ng/(cm^2·Hz). The theoretical mass of bacterial cells adhering to the sensor surface as predicted by the Sauerbrey equation is shown in Figure S-1. We then converted the calculated bacterial cell density derived from our microscopy images into the actual bacterial mass by using the mass of a single *E. coli* bacterial cell (~1 pg) provided by the supplier.

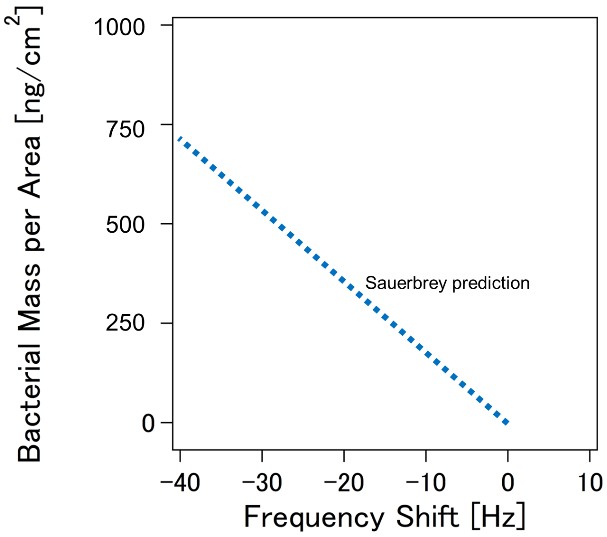

Figure S1. The theoretical mass of bacterial cells adhering onto the sensor surface as predicted by the Sauerbrey equation.

S2 Time progression of bacterial layer viscoelasticity from *D* vs *f* plots

The QCM-D can monitor the changes in the viscoelastic properties of the adhered mass based on the ratio of the energy dissipation and the resonant frequency shift of the oscillating sensor. The energy dissipation is calculated from the decay time of the evanescent shear wave due to the damping caused by the adlayer deposited onto the sensor surface.[S2] A small dissipation is commonly observed in hard and rigid layers since they form stronger bonds to the sensor and thus, only produce a small reduction in the sensor oscillation. The opposite is true for soft and viscous layers where sensor frequency damping is more prominent. [S3,S4]

By plotting the energy dissipation against the frequency shift, it is possible to visualize the changes in the viscoelasticity of the adhered bacterial cell layers through the time course of reversible and irreversible attachment, leading to mature biofilm formation. Figure S-2 shows the changes in the viscoelasticity of the layers for all types of model surfaces investigated in this study.

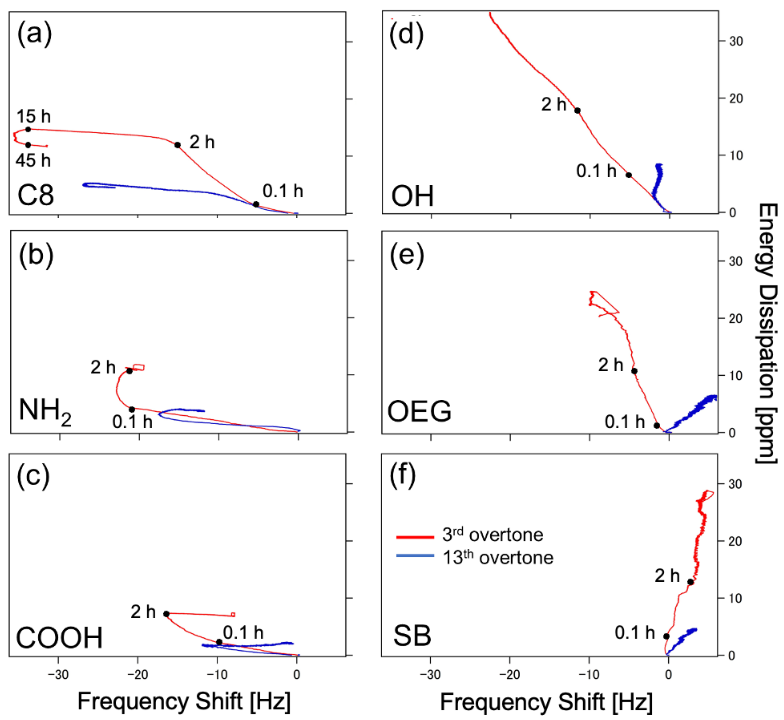

Figure S2. The *D* versus *f* shift plot at $n = 3$ (red) and $n = 13$ (blue) showing the time progression of bacterial adhesion for (a) hydrophobic (C8) SAMs; hydrophilic protein-adsorbing (b) NH_2, (c) COOH, and (d) OH SAMs; and hydrophilic protein-resisting (e) OEG and (f) SB SAMs. Time stamps were shown for the *D* versus *f* shift plot at $n = 3$ to aid time visualization.

S3 Overtone dependence of the equilibrium f shifts

The penetration depths (δ) of the acoustic shear waves are inversely proportional to the overtone number (n), i.e., smaller overtones have larger penetration depths and vice versa. Multiple odd overtones of varying δ can be simultaneously excited by the QCM, and the differences in δ allow the observation of both negative and positive *f* shifts in the system, specifically for micron-sized particles tethered to the resonator via small connections.[S5] For these systems, positive *f* shifts commonly occur at high overtones (n) with shallower δ. [S6] The separation between *f* dominated by

inertia (lower *n*) and those dominated by the small connection (higher *n*) can be estimated by determining the point where the *f* shifts change signs. This point is referred to as the frequency of zero-crossing or f_{ZC}. [S7] For some surfaces investigated (i.e., hydrophobic and hydrophilic protein-adsorbing SAMs except OH SAMs), pure negative shifts were observed which means that the bacterial attachment follows the trend predicted by the Sauerbrey relation. On the other hand, positive shifts were observed for OH SAMs hydrophilic protein-resisting SAMs at high overtone numbers. The plot of the frequency shifts against the overtone number (and hence, δ) is shown in Figure S-3.

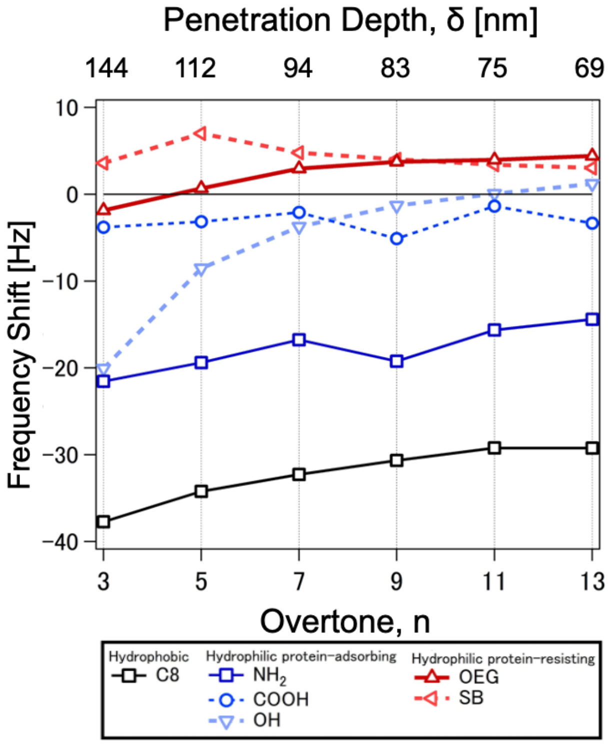

Figure S3. Overtone dependence of the equilibrium *f* shifts for hydrophobic protein-adsorbing (black), hydrophilic protein-adsorbing (blue), and hydrophilic protein-resisting (red) SAMs.

References

(S1) Sauerbrey, G. Verwendung von Schwingquarzen Zur Wägung Dünner Schichten Und Zur Mikrowägung. *Zeitschrift für Physik* **1959**, *155* (2), 206–222. https://doi.org/10.1007/BF01337937.

(S2) Boujday, S.; Briandet, R.; Salmain, M.; Herry, J.-M.; Marnet, P.-G.; Gautier, M.; Pradier, C.-M. Detection of Pathogenic Staphylococcus Aureus Bacteria by Gold Based Immunosensors. *Microchim. Acta* **2008**, *163* (3), 203. https://doi.org/10.1007/s00604-008-0024-3.

(S3) Poitras, C.; Tufenkji, N. A QCM-D-Based Biosensor for E. Coli O157:H7 Highlighting the Relevance of the Dissipation Slope as a Transduction Signal. *Biosens. Bioelectron.* **2009**, *24* (S7), 2137–2142. https://doi.org/10.1016/j.bios.2008.11.016.

(S4) Easley, A. D.; Ma, T.; Eneh, C. I.; Yun, J.; Thakur, R. M.; Lutkenhaus, J. L. A Practical Guide to Quartz Crystal Microbalance with Dissipation Monitoring of Thin Polymer Films. *J. Polym. Sci. A* **2021**, No. pol.20210324. https://doi.org/10.1002/pol.20210324.

(S5) van der Westen, R.; Sharma, P. K.; De Raedt, H.; Vermue, I.; van der Mei, H. C.; Busscher, H. J. Elastic and Viscous Bond Components in the Adhesion of Colloidal Particles and Fibrillated Streptococci to QCM-D Crystal Surfaces with Different Hydrophobicities Using Kelvin-Voigt and Maxwell Models. *Phys. Chem. Chem. Phys.* **2017**, *19* (37), 25391–25400. https://doi.org/10.1039/c7cp04676f.

(S6) Olsson, A. L. J.; van der Mei, H. C.; Johannsmann, D.; Busscher, H. J.; Sharma, P. K. Probing Colloid-Substratum Contact Stiffness by Acoustic Sensing in a Liquid Phase. *Anal. Chem.* **2012**, *84* (10), 4504–4512. https://doi.org/10.1021/ac300366s.

(S7) Johannsmann, D.; Langhoff, A.; Leppin, C. Studying Soft Interfaces with Shear Waves: Principles and Applications of the Quartz Crystal Microbalance (QCM). *Sensors* **2021**, *21* (10). https://doi.org/10.3390/s21103490.
